# Hospital Meetings

**Published:** 1903-02-28

**Authors:** 


### HOSPITAL MEETINGS.

### CHARING CROSS HOSPITAL.

The Earl of Kilmorey, K.P., presided over the eighty-second annual Court of Governors of Charing Cross Hospital on the 18th inst. After the usual formalities, the Chairman touched upon the chief points of interest in the report. The committee, he said, had a right to congratulate itself on the installation of the nurses in their new home. Great progress had also been made with the grand staircase, with four isolation wards above it, and the new out-patient and casualty departments, and the new wards and quarters for the resident staff in King William Street were making good progress. This portion of the work would be much expedited when the nurses' old house had been cleared away. It had been found necessary to make some alteration in the plans, which would not only be a saving of expense, but which would also hasten the completion of the entire work by a twelvemonth. Considerable doubt had arisen as to whether the walls of the old building would support the weight of the new operating theatres and laboratories which it was proposed to build on the top floor, where the nurses' bedrooms used to be, and it was found that it would be necessary to underpin and strengthen them in other ways, thus involving considerable expenditure. It had therefore been decided to put the theatres, etc., on the King William Street block, and to devote half the top floor of the Agar Street block to servants' bedrooms. The sum of £5,477 was raised at the Festival dinner, at which his Grace the Duke of Fife presided, whose excellent and moving speech was very striking. The Duke of Fife had certainly made himself thoroughly acquainted with the statistics. He could say with confidence that the interest and co-operation of the President and other members of the Royal Family was a source of strength to the committee, and he was sure it would continue to be so. Mr. Chamberlain, whose unfortunate accident had been treated in the hospital, had very kindly sent a contribution of 50 guineas. There had been a slight increase in the annual subscriptions, which were £2,319 in 1901 and £2,411 in 1902; and the hospital was greatly indebted to the Ladies' Committee for their valuable services in promoting various entertainments in aid of the funds. The series of Living Pictures at the Savoy Hotel, designed and arranged by Messrs. G. J. Frampton, R.A., J. J. Shannon, A.R.A., and J. M. Swan, A.R.A., brought in £638. The resignation of Miss H. A. C. Gordon through ill-health was sincerely regretted: she organised the nursing staff in 1889, when the Council took the nursing into their own hands. Previously it was managed by St. John's House. The appointment of Miss Mildred Heather-Bigg, formerly a sister in Golding Ward, and lately Matron of the Chelsea Hospital for Women, was a matter for congratulation: her qualifications and experience eminently suited her for the post. "Finally," the Chairman concluded, "we have triumphantly broken down the dislike of Tommy Atkins to convalescent homes. Seventy soldiers invalided home were received at the Convalescent Home at Walton-on-Thames, and they appear greatly to have enjoyed their stay."

The Chairman's last word, as might be expected, was on the financial affairs of the hospital, which are causing considerable anxiety. The past year has been unfavourable for obtaining money, and 1903 opened with a deficit of £14,000. The amount required for the maintenance of the building and convalescent home is £17,000, of which £15,000 has to be raised from benevolent sources. In 1902 the extraordinary expenditure amounted to £33,000, and it is not likely that a less sum will be required this year, making a total of £62,000. The most strenuous efforts were therefore urged in order that this sum might be raised, and the hospital cleared of debt.

The report and accounts were adopted, and the meeting closed with the usual votes of thanks.

### THE LONDON FEVER HOSPITAL.

The annual meeting of this hospital was held on Friday, the 20th inst., at the Hotel Victoria. The Lord Balfour of Burleigh presided. The financial statement was adopted without comment, and the adoption of the report for 1902, read by Dr. Sidney Phillips, was moved by Sir William H. Broadbent, who congratulated the medical staff on the extremely small mortality both in diphtheria (6·4) and in scarlet fever (1·3) cases.

The Chairman (Lord Balfour) said that during 1902 there were 234 patients less than in the previous year, 669 being treated. The relief to the hospital was very acceptable, as it gave opportunity for thorough cleaning and disinfecting. It did not, however, diminish the expense, as the staff of nurses had to be retained at something like its normal strength, to be in readiness for arrivals. Of the 599 new patients admitted, 401 paid a fee of £3 3s.; eight were treated free or at a reduction; 166 free; and 24 were in private rooms: these paid practically the full cost. Altogether the patients paid fees amounting to one-seventh of the whole cost, the other six-sevenths falling as a charge on the funds. He thought public opinion approved the principle of payment by patients who could afford it, and it was a testimony to the work of this hospital that many more sufferers during the last few years had actually become donors or annual subscribers, thus showing their appreciation of the benefits received. After speaking of the kindness of the Lord Mayor and Lady Mayoress in organising the reception at the Mansion House to celebrate the centenary of the hospital, and of the Duke and Duchess of Argyll in attending, the chairman said it was gratifying to know that the Prince of Wales had headed the subscription list with a second donation. Purely as a consequence of the centenary, the donations showed an increase of £2,494 (being £4,266), but there was a falling off of £34 in annual subscriptions, which amounted to £4,808. He could not look upon this as a serious diminution; it was, however, the first time for 20 years that there had been a falling off at all. It showed how carefully an institution of this kind required watching to prevent a wasting away of subscriptions. Considering the many rival claims, the hospital did not come off badly last year. King Edward's Fund had been generous in its treatment, the grant last year being £1,500, of which £1,000 was to be spent on providing a convalescent home. That was a matter constantly before the committee, but he was afraid the home was still in the future. The committee did not see their way towards making a move until the hospital was richer than at present. Reconstruction of the hospital had also been urged both by the medical advisers and by the council of King Edward's Fund; that also was still under consideration. A large and comprehensive scheme of rebuilding was before the committee, who were anxious to see it carried out; the want of money was, however, the difficulty. They could only make the want known, and trust. The speaker concluded with an allusion to the peaceful and harmonious working of the committee and staff, and the usual votes of thanks closed the proceedings.

### THE CITY OF LONDON LYING-IN HOSPITAL.

Mr. J. Francis was voted to the chair at the annual meeting of this hospital, which took place on the 18th inst.

There was one item, the chairman said, that had militated to a certain extent against the work of the past year, namely, the state of the building. There was a report that it would be closed, and this had affected the number of patients, which was 126 less than in 1901. There was, however, a considerable increase in the out-patients' department, where 2,242 cases were treated in their own homes. The training school maintained its high state of efficiency under Miss Fox; 47 midwives and 127 monthly nurses were trained during the year, and all but two candidates received the L.O.S. certificate. The accounts showed an income amounting to £4,631, and there was a balance of £446 on the right side. The report of the medical staff was read by the secretary, and both were adopted. The seconder of the motion alluded to an action now pending with regard to the undermining of the hospital by the railway; he hoped the issue would be satisfactory from the point of view of the hospital. The building is temporarily supported on the outside to make it secure.

